#  Perfil sociodemográfico das pessoas em situação de rua notificadas com tuberculose no Município do Rio de Janeiro, Brasil, nos anos de 2015 a 2019

**DOI:** 10.1590/0102-311XPT051122

**Published:** 2023-10-13

**Authors:** Janaína Rosenburg Gioseffi, Sandra Mara Silva Brignol, Guilherme Loureiro Werneck

**Affiliations:** 1 Instituto de Estudos em Saúde Coletiva, Universidade Federal do Rio de Janeiro, Rio de Janeiro, Brasil.; 2 Instituto de Saúde Coletiva, Universidade Federal da Bahia, Salvador, Brasil.; 3 Instituto de Medicina Social, Universidade do Estado do Rio de Janeiro, Rio de Janeiro, Brasil.

**Keywords:** Tuberculose, Pessoas em Situação de Rua, Vulnerabilidade Social, Tuberculosis, Homeless Persons, Social Vulnerability, Tuberculosis, Personas en Situación de Calle, Vulnerabilidad Social

## Abstract

O Brasil é um dos 30 países com maior incidência de tuberculose (TB). Pessoas em situação de rua (PSR) têm 56 vezes mais riscos para o adoecimento do que a população geral por terem menor renda e acesso à saúde. Os objetivos do estudo foram apresentar o perfil sociodemográfico e epidemiológico de PSR notificadas para TB entre 2015 e 2019 na cidade do Rio de Janeiro e analisar relações entre as variáveis estudadas e desfechos da TB. Trata-se de estudo transversal com dados secundários das notificações de TB em PSR no período e local do estudo. Foi realizada análise descritiva, seguida da verificação de associação entre variáveis selecionadas e desfechos para TB, com teste qui-quadrado e regressão logística multinomial, para obtenção da razão de chances (OR). O perfil predominante das PSR com TB é de homens (74,9%), negros (76,2%), com idade média de 43,3 anos (DP = 12,0) e faixa etária entre 30 e 59 anos (78,5%). O desfecho mais frequente foi abandono do tratamento (43,3%), seguido por cura (29,9%) e óbito (3,6%). As análises mostraram que raça negra (OR = 1,4; IC95%: 1,1-1,9) e uso de drogas (OR = 1,7; IC95%: 1,3-2,3) e álcool (OR = 1,3; IC95%: 1,0-1,7) foram fatores de risco para abandono do tratamento, enquanto faixas etárias a partir de 30 anos (OR = 0,7; IC95%: 0,5-0,9) e forma extrapulmonar (OR = 0,2; IC95%: 0,1-0,6) foram aspectos de proteção. A vulnerabilidade das PSR se particulariza em perfis de raça e gênero, tal qual a TB, portanto, é necessário reforçar ações de prevenção e tratamento efetivas para aumentar o acesso aos serviços de saúde e o enfrentamento da TB nesse contexto, além de atentar para a alta proporção de dados incompletos que limitam as análises desse agravo.

## Abstract (en)

Brazil is one of the 30 countries with the highest incidence of tuberculosis (TB) and homeless people (HP) have 56 times more risk for illness than the general population, due to lower income and access to health. This study aimed to present the sociodemographic and epidemiological profile of HP notified for TB from 2015 to 2019 in the municipality of Rio de Janeiro and to analyze relationships between the variables studied and TB outcomes. This is a cross-sectional study with secondary data on TB notifications in HP in the period and place of the study. Descriptive analysis was performed, followed by analysis of the association between selected variables and TB outcomes with chi-square test and multinomial logistic regression to obtain the odds ratio (OR). The profile of HP with TB is men (74.9%), blacks (76.2%), with a mean age of 43.3 years (SD = 12.0), aged from 30 to 59 years (78.5%). The most frequent outcome was treatment withdrawal (43.3%), followed by cure (29.9%), and death (3.6%). The analyses showed that black individuals (OR = 1.4; 95%CI: 1.1-1.9), drug (OR = 1.7; 95%CI: 1.3-2.3) and alcohol use (OR = 1.3; 95%CI: 1.0-1.7) were risk factors for treatment withdrawal and that age groups older than 30 years or older (OR = 0.7; 95%CI: 0.5-0.9) and the extrapulmonary form (OR = 0.2; 95%CI: 0.1-0.6) were protective factors. The vulnerability of the HP is particularized in racial and gender profiles, as well as TB, thus reinforcing effective prevention and treatment actions is necessary to increase access to health services and the fight against TB in this context. And pay attention to the high proportion of incomplete data that limit the analyses for this problem.

## Resumen

Brasil es uno de los treinta países con mayor incidencia de tuberculosis (TB), y las personas en situación de calle (PSC) tienen 56 veces más riesgo de contraer la enfermedad que la población en general debido a menores ingresos y acceso a la salud. El objetivo de este estudio fue presentar el perfil sociodemográfico y epidemiológico de las PSC diagnosticas con TB entre 2015 y 2019 en la ciudad de Río de Janeiro, así como analizar las relaciones entre las variables estudiadas y los resultados de la TB. Se trata de un estudio transversal, con datos secundarios de los casos de TB en las PSC para el período y el lugar del estudio. Se realizó el análisis descriptivo, seguido del análisis de la asociación entre las variables seleccionadas y los resultados para TB con la aplicación de la prueba de chi-cuadrado y de regresión logística multinomial para obtener el *odds ratio* (OR). En el perfil de las PSC con TB se encuentran hombres (74,9%) negros (76,2%), de edad promedio de 43,3 años (DE = 12,0), en el grupo de edad de entre 30 y 59 años (78,5%). El resultado más frecuente fue el abandono del tratamiento (43,3%), seguido de la cura (29,9%) y la muerte (3,6%). Los análisis mostraron que la raza negra (OR = 1,4; IC95%: 1,1-1,9), el consumo de drogas (OR = 1,7; IC95%: 1,3-2,3) y el alcohol (OR = 1,3; IC95%: 1,0-1,7) fueron los factores de riesgo para el abandono del tratamiento, y que los grupos de edad de más de 30 años (OR = 0,7; IC95%: 0,5-0,9) y la forma extrapulmonar (OR = 0,2; IC95%: 0,1-0,6) fueron los factores protectores. La vulnerabilidad de las PSC se agudiza en los perfiles de raza y de género tal como la TB, por lo que es necesario fortalecer las acciones efectivas de prevención y tratamiento para aumentarles el acceso a los servicios de salud y el combate de la TB en este contexto. Y, además, prestar atención a la alta proporción de datos incompletos que limitan los análisis para esta condición.

## Introdução

A tuberculose (TB) é uma doença infecciosa de grande relevância para a saúde pública mundial ^1^. Trata-se da infecção gerada pelo agente *Mycobacterium tuberculosis*, uma bactéria transmitida por aerossóis ^1^ que provoca uma infecção granulomatosa em seu hospedeiro ^1^^,^^2^. A TB está entre as doenças que mais levam a óbito no mundo atualmente ^1^.

O Brasil se posiciona entre os 30 países com maiores incidências da doença ^2^ e se comprometeu, durante a Assembleia Mundial de Saúde da Organização Mundial da Saúde (OMS), em 2017, a aderir à estratégia global de enfrentamento à TB por meio da elaboração do Plano Nacional pelo Fim da Tuberculose como Problema de Saúde Pública, pela Coordenação-Geral do Programa Nacional de Controle da Tuberculose (CGPNCT) ^3^. Esse plano se compromete com as metas da OMS de eliminar a TB como problema mundial de saúde pública, incluindo diminuir a incidência da TB para menos de 10 casos por 100 mil habitantes e reduzir o número de óbitos em pelo menos 95% até 2035 ^3^.

No entanto, mesmo passados cinco anos, o Brasil permanece entre os países com piores indicadores para o agravo, com incidência de 31,6 casos novos por 100 mil habitantes em 2020 e 4,5 mil óbitos em números absolutos em 2019 ^4^.

Historicamente, a TB na cidade do Rio de Janeiro se apresenta como a maior responsável pela mortalidade na população, sobretudo entre o fim do século XIX e início do século XX, época em que os mais acometidos eram indivíduos de menor renda e menor acesso à saúde ^5^. No ano de 2020, a capital do Rio de Janeiro teve a segunda maior incidência entre os estados da Federação, representando 84,9 novos casos por 100 mil habitantes ^4^.

Na rede pública de saúde no município, o cuidado e controle da TB em toda a população são realizados pela atenção básica, com acompanhamento dos pacientes e tratamento supervisionado, além de contar com o Laboratório Central de Saúde Pública Noel Nutels (Lacen/RJ) e o apoio de diversas instituições acadêmicas para ações que vão desde o diagnóstico até a atenção terciária, como no caso de acompanhamento de casos de TB resistentes ^6^^,^^7^.

Porém, a cobertura geral pela atenção básica na cidade do Rio de Janeiro foi de 47,29% em abril de 2020 e a da Estratégia Saúde da Família (ESF) foi de 40,96% no mesmo período, segundo o Sistema de Informação e Gestão da Atenção Básica (e-Gestor) do Ministério da Saúde ^8^. Quando comparadas às coberturas nacionais - atenção básica (76,5%) e ESF (65,36%) -, as coberturas desses serviços de saúde na cidade estão distantes do necessário para promover um bom alcance na universalização da saúde pública aos seus cidadãos ^8^.

Deve-se considerar que a TB acomete principalmente populações dos estratos socioeconômicos com menor poder aquisitivo e, consequentemente, em condições de vida mais precarizadas e vulnerabilizadas ^2^^,^^9^^,^^10^^,^^11^: moradores das favelas, pessoas privadas de liberdade e pessoas em situação de rua (PSR).

As PSR são caracterizadas como indivíduos com enfraquecimento de suas relações sociais e familiares, em situação de extrema pobreza e inexistência de moradia regular convencional, usando espaços públicos como sua moradia e sustento, bem como albergues e abrigos, de forma permanente ou temporária ^12^. Elas têm suas vidas precarizadas ao extremo, distantes da percepção social de direitos humanos e cidadania. Mesmo com políticas específicas para essa população, como a Política Nacional para a Inclusão Social da População em Situação de Rua ^13^, sua aplicabilidade fica aquém do necessário, e as PSR permanecem excluídas de atuações institucionais e sociais, em particular dos serviços de saúde.

Nessa população, as doenças mais prevalentes entre transmissíveis e crônicas não transmissíveis são: TB, HIV e aids, hipertensão arterial e diabetes, além de distúrbios psicossociais advindos do abuso de drogas e álcool ^13^. A TB nas PSR alcança incidências muito altas em todo o mundo ^14^^,^^15^, podendo chegar a mais de 500 novos casos por 100 mil habitantes ^14^^,^^16^. No Brasil e entre os estados, é difícil calcular a incidência da TB nessa população, uma vez que não são realizados sistematicamente censos estaduais e nacionais para a contagem das PSR ^17^, porém sabe-se que elas têm 56 vezes mais riscos para o adoecimento por TB e representam uma carga de 2,6% entre novos casos para o agravo no Brasil ^18^.

As dificuldades inerentes à vida na rua, o preconceito e a insegurança fazem com que a adesão aos tratamentos de saúde seja menor entre PSR do que na população em geral ^19^.

A iniciativa do Consultório na Rua, implementado na atenção básica de saúde brasileira em 2011 ^20^, é uma tentativa de promover o acesso aos serviços de saúde às PSR sem que elas passem por constrangimentos ou estigmas ao procurarem as unidades de atenção básica. Conta atualmente com o financiamento federal de 307 equipes de Consultório na Rua, com distribuição relacionada ao número de habitantes por cidade: 122 equipes para municípios com mais de 300 mil habitantes e 185 para municípios entre 100 e 300 mil habitantes ^21^.

No Município do Rio de Janeiro, percebe-se a falta de uniformidade na distribuição da estratégia Consultório na Rua entre suas regiões. Entre as 10 áreas da coordenação de atenção primária existentes, apenas seis contam com equipes para esse tipo de atendimento: Centro, Benfica, Jacarezinho, Acari, Realengo e Paciência ^22^. Além do acesso ao Consultório na Rua, uma pesquisa realizada pela Secretaria Municipal de Assistência Social e Direitos Humanos no Rio de Janeiro mostrou entre seus resultados que aproximadamente metade das PSR entrevistadas não tiveram acesso a uma unidade de saúde no ano de 2018 ^23^.

Existe uma grande lacuna de produções científicas em saúde para essa população, inclusive de dados oficiais sobre incidência e mortalidade por TB nas PSR. Portanto, os benefícios esperados do artigo são que os resultados possam ser usados por pesquisadores para subsidiar hipóteses para suas futuras pesquisas e por gestores dos serviços de saúde, responsáveis pela formulação e implantação de políticas públicas, tomada de decisão, melhora da qualidade do atendimento das PSR nos serviços de saúde, como subsídios para políticas sociais e de saúde voltadas para a população do estudo.

Nesse contexto, os objetivos deste artigo foram definir o perfil sociodemográfico e epidemiológico de PSR notificadas para TB entre os anos de 2015 e 2019 no Município do Rio de Janeiro e analisar possíveis relações entre fatores de risco e desfechos da TB.

## Metodologia

Trata-se de um estudo do tipo transversal com dados secundários sobre a ocorrência de TB entre PSR no período de janeiro de 2015 a dezembro de 2019 no Município do Rio de Janeiro. Os dados foram provenientes do Sistema de Informação de Agravos de Notificação (SINAN NET, versão 5.0), cedidos pela Secretaria Municipal de Saúde do Rio de Janeiro (SMS/RJ).

A população do estudo foi definida como todos os registros notificados referentes aos casos de TB nas PSR no período e local do estudo, os quais foram entregues à autora no início de 2021 pela SMS/RJ.

Os anos de estudo foram escolhidos por corresponderem à maior quantidade de registros atuais no SINAN, uma vez que a categoria de “população em situação de rua” só foi implementada a partir de 2014 na ficha de notificação e tabulação dos dados no Departamento de Informática do SUS (DATASUS). Ainda assim, após analisar o conjunto de registros de 2014 a 2019 (2.198 notificações), foram descartadas as notificações do ano de 2014 (n = 197) por apresentarem inconsistências no preenchimento de variáveis, além de muitos campos ignorados ou não preenchidos. O total de registros para as análises foi de 2.001 notificações.

Após a organização e limpeza do banco de dados, foram selecionadas as seguintes variáveis para análise: desfecho (cura, abandono, óbito por TB, óbito por outras causas, transferência, mudança de diagnóstico, mudança de esquema, falência, abandono primário e ignorado); sexo (feminino e masculino); raça/cor (preta, parda, branca, amarela e indígena); HIV (negativo, positivo, em andamento e não realizado); forma (pulmonar, extrapulmonar e ambas); data de notificação (data de nascimento); consumo de álcool (sim, não e ignorado); uso de tabaco (sim, não e ignorado); uso de drogas ilícitas (sim, não e ignorado); tratamento diretamente observado - TDO (sim, não e ignorado); e código do bairro de notificação.

A partir da “data de notificação” do agravo, o ano foi isolado e foi criada a variável “ano de notificação”, a fim de permitir a análise da variação de número de casos anuais e a construção da série histórica do período. Para a idade, foi criada a variável “faixa etária” a partir da “data de notificação” subtraída da variável “data de nascimento”, resultando na idade em anos, que posteriormente foi categorizada em 0-29 anos, 30-59 anos e 60 anos ou mais. A seguir, para as variáveis que apresentavam mais que quatro categorias e com frequência relativa abaixo de 5%, foi realizada a agregação das categorias para viabilizar a análise de modelagem estatística, resultando nas variáveis “raça/cor” (branca, negra - agregada de preta e parda -, outra, ignorado) e “desfecho da TB” (cura, abandono, óbito por TB, outro). Dados faltantes nas variáveis foram organizados na categoria “ignorado” de cada variável.

A análise estatística descritiva (medidas resumo) precedeu a análise da associação entre as variáveis independentes e o desfecho para TB (cura, abandono, óbito, outro) - variável dependente. O teste qui-quadrado e/ou teste exato de Fisher com nível de significância de 5% foi usado para testar a hipótese de independência entre o desfecho (infecção para TB) e as variáveis independentes (variáveis sociodemográficas e epidemiológicas). Posteriormente, efetuou-se a regressão logística multinomial, indicada para desfechos com mais de duas categorias, para obtenção da razão de chances (OR), medida de associação e seus respectivos intervalos de 95% de confiança (IC95%) ^24^. Após uma avaliação da frequência relativa, optou-se pela retirada da categoria “ignorado” de todas as variáveis antes da modelagem estatística. A limpeza do banco de dados e análises foram realizadas no programa estatístico RStudio, versão 1.2.5001 (https://rstudio.com/). O programa TerraView, versão 4.2.2 (http://www.dpi.inpe.br/terraview), foi utilizado para produzir o mapa da distribuição geográfica do número de casos no período, segundo os bairros do município.

O estudo foi cadastrado na Plataforma Brasil (https://conselho.saude.gov.br/plataforma-brasil-conep) e aprovado pelo Comitê de Ética em Pesquisa do Hospital Universitário Antônio Pedro da Universidade Federal Fluminense (CAAE 25832719.6.0000.5243 e parecer nº 3.758.351). A pesquisa também foi aprovada pelo Comitê de Ética em Pesquisa da SMS/RJ (CAAE 25832719.6.3001.5279 e parecer nº 4.034.26).

## Resultados

Do total de 2.001 notificações entre 2015 e 2019, o perfil das PSR com TB foi marcado por predominância do sexo masculino (74,9%) e da raça/cor negra (76,2%). Foi observado o total de 151 dados ignorados para raça/cor (7,5%) (Tabela 1).

**Tabela 1:** Tabela de contingência para análises bivariadas e regressão logística multinomial.

Variáveis	Cura	Abandono			Óbito			Outros	Total	Valor de p
	n (%)	n (%)	OR	IC95%	n (%)	OR	IC95%	n (%)	n (%)	
Total	598 (29,9)	867 (43,3)			73 (3,7)			463 (23,1)	2.001 (100,0)	
Raça/Cor										< 0,001
Branca	121 (20,2)	118 (13,6)	1,0		7 (9,6)	1,0		62 (13,4)	308 (15,4)	
Negra	439 (73,4)	681 (78,5)	1,4	1,1-1,9	58 (79,5)	2,9	0,8-10,0	347 (74,9)	1.525 (76,2)	
Outra	9 (1,5)	7 (0,8)	1,1	0,4-2,9	0 (0,0)	-	-	1 (0,2)	17 (0,8)	
Ignorada	29 (4,8)	61 (7,0)	-		8 (11,0)	-	-	53 (11,4)	151 (7,5)	
Sexo										0,082
Feminino	152 (25,4)	237 (27,3)	1,0		14 (19,2)	1,0		100 (21,6)	503 (25,1)	
Masculino	446 (74,6)	630 (72,7)	1,0	0,8-1,3	59 (80,8)	1,2	0,5-3,0	363 (78,4)	1.498 (74,9)	
Faixa etária (anos)										< 0,001
0-29	126 (21,2)	218 (25,3)	1,0		9 (12,5)	1,0		81 (17,7)	434 (21,9)	
30-59	425 (71,4)	617 (71,7)	0,8	0,6-1,1	51 (70,8)	0,8	0,3-1,9	347 (75,9)	1.440 (72,5)	
60 ou mais	44 (7,4)	26 (3,0)	0,4	0,2-0,7	12 (16,7)	0,6	0,2-4,6	29 (6,3)	111 (5,6)	
Forma										< 0,001
Pulmonar	570 (95,3)	842 (97,1)	1,0		70 (95,9)	1,0		434 (93,7)	1.916 (95,8)	
Extrapulmonar	21 (3,5)	8 (0,9)	0,2	0,1-0,6	0 (0,0)	-	-	19 (4,1)	48 (2,4)	
Pulmonar e extrapulmonar	7 (1,2)	17 (2,0)	0,8	0,3-2,1	3 (4,1)	-	-	10 (2,2)	37 (1,8)	
HIV										< 0,001
Negativo	481 (80,4)	642 (74,0)	1,0		51 (69,9)	1,0		305 (65,9)	1.479 (73,9)	
Em andamento	2 (0,3)	5 (0,6)	-	-	0 (0,0)	-	-	15 (3,2)	22 (1,1)	
Positivo	81 (13,5)	134 (15,5)	1,2	0,9-1,6	7 (9,6)	2,1	0,9-5,4	109 (23,5)	331 (16,5)	
Não realizado	34 (5,7)	86 (9,9)	-	-	15 (20,5)	-	-	34 (7,3)	169 (8,4)	
Uso de tabaco										< 0,001
Não	251 (42)	320 (36,9)	1,0		19 (26,0)	1,0		173 (37,4)	763 (38,1)	
Sim	297 (49,7)	453 (52,2)	0,8	0,6-1,1	30 (41,1)	2,3	0,9-5,8	206 (44,5)	986 (49,3)	
Ignorado	50 (8,4)	94 (10,8)	-	-	24 (32,9)	-	-	84 (18,1)	252 (12,6)	
Uso de drogas										< 0,001
Não	197 (32,9)	184 (21,2)	1,0		21 (28,8)	1,0		123 (26,6)	525 (26,2)	
Sim	363 (60,7)	607 (70,0)	1,8	1,4-2,4	27 (37,0)	0,5	0,2-1,3	267 (57,7)	1.264 (63,2)	
Ignorado	38 (6,4)	76 (8,8)	-	-	25 (34,2)	-	-	73 (15,8)	212 (10,6)	
Consumo de álcool										< 0,001
Não	347 (58,0)	421 (48,6)	1,0		20 (27,4)	1,0		189 (40,8)	977 (48,8)	
Sim	213 (35,6)	364 (42,0)	1,3	1,0-1,6	32 (43,8)	2,1	0,9-4,6	200 (43,2)	809 (40,4)	
Ignorado	38 (6,4)	82 (9,5)	-	-	21 (28,8)	-	-	74 (16,0)	215 (10,7)	
TDO										< 0,001
Não	122 (20,4)	166 (19,1)	1,0		12 (16,4)	1,0		63 (13,6)	363 (18,1)	
Sim	385 (64,4)	504 (58,1)	0,9	0,7-1,2	24 (32,9)	1,2	0,5-3,0	213 (46,0)	1.126 (56,3)	
Ignorado	91 (15,2)	197 (22,7)	-	-	37 (50,7)	-	-	187 (40,4)	512 (25,6)	

IC95%: intervalo de 95% de confiança; OR: razão de chances; TDO: tratamento diretamente observado.

A média de idade foi de 39,3 anos (desvio padrão - DP = 11,9), sendo de 35,1 anos (DP = 10,1) para mulheres e 40,7 anos (DP = 12,2) para homens. A faixa etária predominante foi 30-59 anos (72,5%). Os resultados foram calculados segundo a frequência e os percentuais ajustados, portanto, os números podem variar devido a dados faltantes.

A forma de manifestação mais comum da TB foi a pulmonar (95,8%). A infecção pelo HIV foi detectada em 16,5% das PSR notificadas com TB. Os usuários de tabaco e drogas ilícitas representaram 49,3% e 63,2% da população em situação de rua com TB, respectivamente, enquanto usuários de álcool totalizaram 40,4%. Quanto ao TDO, 56,3% dos pacientes foram contemplados pelo programa e 18,1% não, devendo-se considerar que dados ignorados dessa variável totalizaram 25,6%.

**Figura 1:**
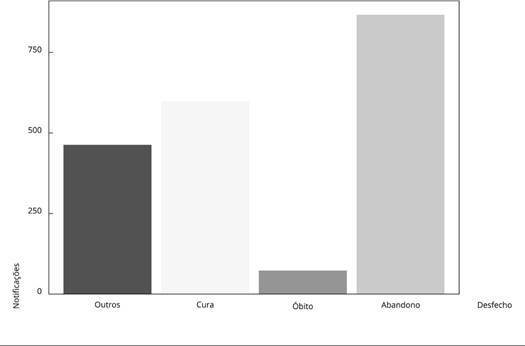
Gráfico de desfechos para tuberculose (TB) em pessoas em situação de rua (PSR). Município do Rio de Janeiro, Brasil, 1º de janeiro de 2015 a 31 de dezembro de 2019.

No período estudado, o desfecho mais frequente foi o abandono do tratamento (43,3%), seguido por cura (29,9%) e óbito por TB (3,7%). Outros desfechos totalizaram 463 observações (23,1%) (Figura 1).

Fonte: Sistema de Informação de Agravos de Notificação ^37^.

A análise bivariada mostrou que cor da pele negra (OR = 1,4; IC95%: 1,1-1,9), uso de drogas (OR = 1,8; IC95%: 1,4-2,4) e de álcool (OR = 1,3; IC95%: 1,0-1,6) foram associados com maior chance de abandono do tratamento. A faixa etária 60 anos ou mais (OR = 0,4; IC95%: 0,2-0,7), assim como manifestar a forma extrapulmonar da doença (OR = 0,2; IC95%: 0,1-0,6), foram relacionadas com menor frequência de abandono do tratamento.

**Figura 2:**
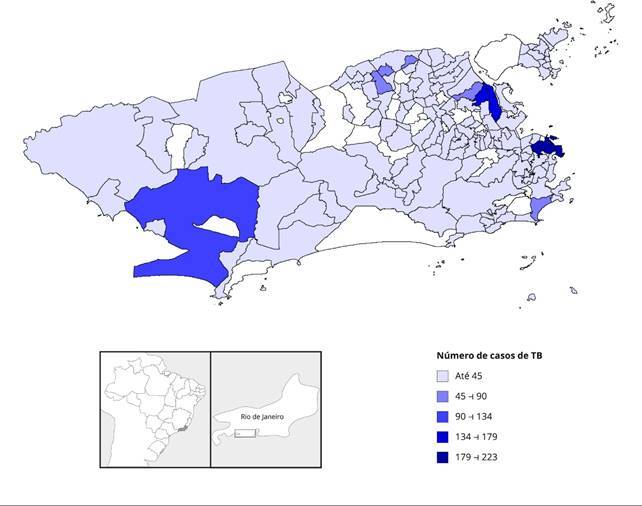
Distribuição dos casos notificados de tuberculose (TB). Município do Rio de Janeiro, Brasil, 2015-2019.

A Figura 2 mostra a distribuição geográfica dos casos notificados. Os bairros com mais notificações foram o Centro (11,7%), seguido por Ramos (8,2%), Complexo da Maré (7,7%) e Pavuna (3,9%) - todos na Zona Norte do município -, além de Guaratiba (5,7%), na Zona Oeste.

**Figura 3:**
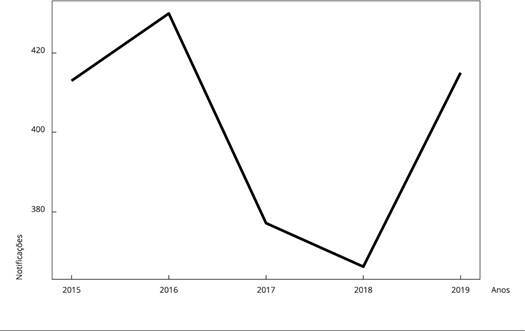
Série histórica das notificações de tuberculose (TB) em pessoas em situação de rua (PSR). Município do Rio de Janeiro, Brasil, 1º de janeiro de 2015 a 31 de dezembro de 2019.

A série histórica (Figura 3) mostra uma grande variação no número de notificações por ano no período estudado, com 413 em 2015, 430 em 2016, 377 em 2017, 366 em 2018 e 415 em 2019.

Fonte: Sistema de Informação de Agravos de Notificação ^37^.

## Discussão

Os resultados encontrados são compatíveis com a literatura ^14^^,^^15^^,^^25^^,^^26^ quanto ao perfil sociodemográfico: homens negros entre 25 e 49 anos e com maior chance de abandonarem o tratamento. Esse perfil está inserido no contexto da necropolítica atual ^27^ - conceito elaborado por Achille Mbembe ^28^ sobre a atuação violenta e estruturalmente racista do Estado nas sociedades contemporâneas pela diferenciação e segregação racial da sua população -, determinada em criar condições para que uma pessoa negra de alguma forma seja mais exposta à TB, seja no sistema prisional ou em situação de habitação precária como as favelas - ambos os contextos sendo reconhecidamente locais de incidência alta para TB ^9^^,^^11^. A ampla maioria dos residentes das favelas no Rio de Janeiro são pessoas pretas e pardas, como mostram os resultados do censo da Maré realizado em 2019 ^29^, com 62,1% dos moradores desse grande complexo assim autodeclarados. Entre as PSR, em que se verifica esse mesmo perfil, há também maior chance de abandono de tratamento para TB entre negros.

Os resultados também mostram prognóstico desfavorável quanto aos desfechos possíveis para o acometimento pela TB, o que condiz com achados de outros estudos realizados recentemente ^14^^,^^15^^,^^17^, principalmente quando comparados à população geral da cidade do Rio de Janeiro ^30^. A alta porcentagem de coinfecção por TB e HIV e a forma de manifestação desse agravo são preocupantes devido à associação desses fatores com desfechos de abandono e óbito ^26^. Isso se dá pela produção de vidas vulnerabilizadas ^31^ e não integráveis aos direitos e à cidadania, que permite que PSR, entre outras populações estigmatizadas e marginalizadas, tenham dificuldade no acesso às políticas públicas de distribuição de renda e benefício e aos serviços de saúde ^19^.

Mesmo não havendo resultados neste estudo sobre a atuação do Consultório na Rua por não constar no SINAN, além do tratamento diretamente observado, é importante comentar que estratégia do Consultório na Rua apresenta boa aceitação pelas PSR ^19^^,^^32^, desempenhando papel fundamental no tratamento de TB, HIV e outras infecções e doenças não transmissíveis.

É pertinente apontar, ainda, que a maior concentração de PSR ocorre em locais onde há maior circulação de transeuntes, como na região central da cidade, área contemplada pela estratégia do Consultório na Rua, bem como na Zona Norte ^22^. Porém, o bairro de Guaratiba, na Zona Oeste do município, que tem alto número de casos segundo os resultados apresentados, segue sem equipes do Consultório na Rua ^22^, demonstrando a invisibilização dessa área da cidade na gestão municipal.

O tratamento diretamente observado de maneira flexível também foi pontuado como importante, pois leva em consideração o fato de serem andarilhos e necessitarem dessa movimentação para conseguirem recursos e alimentação ^19^. Ao mesmo tempo, é necessário que se estabeleça a padronização de condutas protocolares de tratamento, principalmente no que diz respeito à TB multirresistente (MDR-TB), devido à dificuldade de alcance de resultados positivos em relação à adesão ao tratamento da TB ^33^.

Ainda sobre o protocolo de tratamento, sabe-se que a internação de PSR é mais complicada, pois as submete a um conjunto de repressões e regras que comprometem seu estilo de vida, coibindo o uso de tabaco, álcool e drogas ^32^^,^^34^, por exemplo. Os resultados neste estudo mostram que ser usuário de álcool ou de drogas é um fator associado ao abandono do tratamento. Por outro lado, os resultados também evidenciam que há menor chance de abandono do tratamento quando a forma da TB é extrapulmonar, possivelmente por determinar uma condição mais debilitante que a vista na forma pulmonar.

Atualmente, o contexto brasileiro de grave crise econômica, em parte em decorrência da pandemia de COVID-19, afetou diretamente os estratos sociais mais economicamente e socialmente fragilizados. Nesse cenário, houve diminuição da oferta de empregos formais e informais, dificuldade de acesso ao auxílio emergencial e cortes do Governo Federal, com o programa de transferência de renda Bolsa Família, além da instabilidade política, ocasionando aumento da quantidade de PSR e da insegurança alimentar em todo o país ^35^. Nesse contexto, reflexos deletérios nos níveis de prevalência e incidência da TB são plausíveis, implicando grandes desafios para os serviços de saúde, não só em relação ao tratamento da TB, mas também de outros agravos.

## Conclusão

A vulnerabilidade das PSR se particulariza em perfis de raça e gênero, dentro de um padrão já conhecido. Com isso, é necessário reforçar as ações de prevenção e tratamento, tão fundamentais para enfrentar esse contexto da TB. Tendo em vista a dificuldade de acesso aos serviços de saúde por parte das PSR, o Consultório na Rua é uma importante estratégia para o cuidado dessa população, já que realiza assistência integral, promove o vínculo da equipe com a PSR e possibilita a prevenção de doenças e a promoção da saúde. A educação permanente para profissionais de saúde pode ser uma estratégia para manter o padrão de atenção à saúde das PSR e superar estigmas sobre essa situação sob uma perspectiva de humanização no atendimento dessa população ^36^.

Além disso, as notificações da TB estão distribuídas com maiores frequências no centro do município e em alguns bairros da Zona Norte, com grande variação anual do número de casos nos anos da série histórica do período estudado (2015-2019), sem indicações de queda.

É importante chamar a atenção para a alta proporção de dados incompletos, dificultando as análises e indicando uma subnotificação desse agravo. Em particular, identificou-se uma baixa quantidade das notificações em 2014, possivelmente por mudanças no protocolo de notificação e carregamento no SINAN e por atrasos na computação das notificações de TB no DATASUS. Essas questões precisam ser superadas, e os dados regularizados com urgência. Dessa forma, a educação permanente pode ser uma das ferramentas para as equipes de saúde, esclarecendo a importância do adequado preenchimento das variáveis das fichas de notificação do SINAN, pois aponta os indicadores locais dos agravos, os quais permitem que os profissionais atuem de forma diretiva nas fragilidades encontradas.

O acompanhamento do perfil populacional é imprescindível para futuras análises, visto que o aumento da quantidade de PSR durante a pandemia de COVID-19 pode ter alterado as características do perfil sociodemográfico dessa população, uma vez que famílias inteiras perderam sua renda, sustento e abrigo nos últimos dois anos.

Por fim, será fundamental rever as políticas de apoio social e distribuição de renda diante do aumento do número de PSR no período da pandemia e, paralelamente, equipar as unidades de saúde da atenção básica e Consultório na Rua com profissionais capacitados e insumos para o enfrentamento da TB.

### Limitações

Neste artigo há limitações, uma vez que foram utilizados dados secundários em sua realização, apresentando muitas variáveis com baixa adesão no preenchimento pelos setores de atendimento aos pacientes, além de não contemplar a vivência das PSR.
